# Diphtheritic Ophthalmia Treated with Carbolic Acid and Iodine

**Published:** 1873-01

**Authors:** F. C. Hotz

**Affiliations:** Oculist to the Cook County Hospital, Chicago


ARTICLE III.—*Diphtheritic Ophthalmia Treated with Carbolic Acid and Iodine.* By F. C. HOTZ, M.D., Oculist to the Cook County Hospital, Chicago.

Last year I published, in this journal, a number of cases of diphtheria which had been very successfully treated by the local use of a mixture of carbolic acid and iodine:

| | | |
|---|---|---|
| R. | Acid Carbol. Cryst., . . . . . . . | dr. j. |
| | Tinct. Iodine, . . . . . . . . . . | dr. ss. |
| | Aq. Dest., . . . . . . . . . . | dr. v. |
| | Alcohol, . . . . . . . . . . | dr. j. |

Among those cases there was one of diphtheritic ophthalmia of a very malignant character. The use of the above mixture arrested the progress of this inflammation so effectually, that after two days the eye was out of danger, and made a speedy and complete recovery.

These results were so encouraging, and so strengthened my belief of diphtheria being primarily a local affection, that I at once decided to use it again in similar cases; although the best authorities strongly urged in the treatment of diphtheritic ophthalmia, to abstain from all local use of irritating medicines. Their customary treatment, then, was the placing of the patient under good sanitary conditions, the use of ice (or warm poultices), and the liberal administration of mercury. The results of such treatment were, indeed, better than those obtained by energetic cauterization, which was previously so much in vogue. Still, this method of treatment must have been very unsatisfactory if such experienced oculists as Solberg Wells (in his Treatise on the Diseases of the Eye), had to confess " that we have, unfortunately, but little control over the disease during the first period." And just during this first, *i. e.*, diphtheric stage, the cornea is often destroyed, and so many eyes are lost for ever. To make our treatment satisfactory, we must have a remedy which curtails this most dangerous period of the diphtheritic ophthalmia. Now, after all my observations, the mixture of carbolic acid and iodine seemed to be the remedy wished for. It seemed to break up the diphtheritic (fibrinous) exudations—to melt them, as it were—and to induce their gradual reabsorption, without any disintegration of the inflamed tissues.

Fortunately, I may say, the diphtheritic ophthalmia is of rare occurrence in this part of our country, and an opportunity of trying the carbolated iodine mixture was not offered to me till August. Although it is one case only that I have to report, I will publish it to draw again the attention of the profession to a remedy which has given so great satisfaction to my professional friends and myself, and to induce others who may have ampler opportunity for its use to test its efficiency.

*History of the case.* P. W., aged twenty-five years, laborer, was admitted to the eye ward of Cook County Hospital, August 1, 1872. He had had gonorrhœa for several weeks, and had brought some gonorrhœal virus in his eyes. For one week before admission, his left eye suddenly began to inflame very severely, and four days later, his right eye showed the same signs of a violent inflammation. On admission, the eyelids were red, hot, very tender and greatly swollen, but, at the same time, of that peculiar, hard feeling due to a fibrinous infiltration of the soft tissues. This tenderness and stiffness of the lids rendered their eversion extremely difficult and painful. The conjunctiva of the lids was thickened, gray-white, impregnated, and covered by a fibrinous exudation, which could not be removed without pieces of the conjunctiva being torn out. The ocular conjunctiva also thickened, bloodless, yellow-white, like lard. The secretion of the conjunctiva was copious, but a thin, watery fluid, with some gray flakes of fibrine floating in it. There was no trace of the thick, purulent discharge peculiar to the blennorrhœa. The left cornea was completely necrotic; and in the right cornea, a small grayish-yellow cloud was discovered, near the inner margin. On account of this state of the cornea, warm water was used on the eyes, and calomel (half gr. every two hours) was given; but, on August 3d, no improvement could be noticed in the state of the conjunctiva, while the gray-yellow cloud in the right cornea had extended over its whole surface. The carbolated iodine mixture was then penciled over the conjunctival surface of the everted lids twice daily, and a compress, saturated with a diluted solution (thirty drops of the mixture to a teacupful of water) was kept on the eyes. This treatment very soon broke the violence of the inflammation, and operated so successfully that, on August 6th, all traces of diphtheritic exudation had disappeared. The then red and succulent conjunctiva, the swollen, but soft and flabby lids, and the thick, creamy discharge, indicated that the disease had passed over into the second period, *i. e.*, into blennorrhœal inflammation. This readily healed under the use of sol. argent. nitrat. (scr. j to oz. j, aq. dest.), and the conjunctiva recovered its natural appearance. The improvement in the state of the conjunctiva was closely followed by a favorable change in the condition of the right cornea (the left cornea being totally mortified, was, of course, irretrievably lost). The cloud contracted and gathered into a small abscess in the internal half of the cornea, its external half having become perfectly clear and transparent. The abscess broke through the anterior epithelium, and, filling up with new tissue, it healed in a dense, white opacity near the inner margin of the cornea. But as the center of this membrane, opposite the pupil, had regained its full transparency, the patient had very good sight when he was discharged.

